# Machine Learning-Based Quantification of Lateral Flow Assay Using Smartphone-Captured Images

**DOI:** 10.3390/bios15010019

**Published:** 2025-01-04

**Authors:** Anne M. Davis, Asahi Tomitaka

**Affiliations:** 1 Department of Computer and Information Sciences, University of Houston-Victoria, Victoria, TX 77904, USA; 2 Department of Computer Science, Kennesaw State University, Marietta, GA 30060, USA

**Keywords:** machine learning, deep learning, CNN, lateral flow assay

## Abstract

Lateral flow assay has been extensively used for at-home testing and point-of-care diagnostics in rural areas. Despite its advantages as convenient and low-cost testing, it suffers from poor quantification capacity where only yes/no or positive/negative diagnostics are achieved. In this study, machine learning and deep learning models were developed to quantify the analyte load from smartphone-captured images of the lateral flow assay test. The comparative analysis identified that random forest and convolutional neural network (CNN) models performed well in classifying the lateral flow assay results compared to other well-established machine learning models. When trained on small-size images, random forest models excelled CNN models in image classification. Contrarily, CNN models outperformed random forest models in classifying noisy images.

## 1. Introduction

The lateral flow assay is a paper-based portable sensor used in various applications including pregnancy tests, disease diagnostics, and food safety testing. It detects the presence of specific analytes such as biomolecules, disease pathogens, and toxic compounds in food within 5–30 min without lab equipment [1]. A lateral flow assay test strip consists of a sample well where the liquid sample is dispensed, a sample pad where the sample is absorbed, a conjugate pad that contains antibodies, a detection window composed of nitrocellulose membrane with biorecognition elements that displays the control and test lines, and an absorption pad where the sample flow is concluded. These components are held together by using a backing card. The appearance of the control line and test line indicate the proper liquid flow and the presence of sample analytes, respectively [2]. The most recent application of lateral flow assay is the rapid test for COVID-19 which is used to diagnose patients as COVID-19 positive or negative. Although the lateral flow assay test kit is often used for point-of-care disease diagnostics in rural areas and developing countries due to its long shelf life at room temperature and low production cost, its low sensitivity and poor quantification capacity limit its applications. The downside of the current generation of lateral flow assay is its qualitative nature. It is used for primary screening purposes and suffers from high rates of false diagnostics based on visual observation by the naked eye. An example of such a case is when the test strip gives a faint appearance on the test line, which could confuse the patients without assistance from healthcare professionals. Although it is acceptable for some simple applications such as pregnancy test where yes/no (positive/negative) result is sufficient, the misdiagnosis of infectious diseases could cause serious consequences. Quantification of the assay results is critical in providing important information such as the disease progression/prognosis and safety limit for allergenic/toxic/pollutant compounds [3]. In the case of infectious diseases, the quantitative information helps estimate the severity and infectiousness, thus helping determine the therapeutic actions and quarantine measures. It is especially beneficial in rural areas and developing countries where healthcare resources such as the number of clinics and the skills of healthcare providers are limited and experience higher mortality rates from COVID-19 compared to urban areas [4]. Due to insufficient access to more accurate and sensitive lab-based testing, reliable point-of-care testing which provides quantitative insight becomes crucial. An NIH study identified SARS-CoV-2 nucleocapsid(N) protein levels as a prognostic biomarker for patient outcomes, but clinical application is restricted by the lack of point-of-care quantitative testing [5]. Commercially available COVID-19 LFAs, which predominantly detect N-protein, could serve as a solution to address this limitation. The purpose of this study is to evaluate methods for the quantification of the analyte load of LFAs for scenarios where binary results are insufficient, but laboratory testing is a limiting factor.

The quantification studies of lateral flow assay have been conducted primarily by employing sensors, which detect the gold nanoparticles used as the color source in the assay, or color readers combined with image processing techniques. The latter utilizes the assay images captured by devices such as smartphone-mounted readers, smartphone-based reader accessories, and smartphone cameras, and the images are further processed and analyzed to calculate the analyte concentration [6,7]. This approach requires a controlled environment created by portable boxes where lighting conditions and backgrounds are controlled for image acquisition to remove environmental errors [3]. Alternatively, extensive image preprocessing techniques such as object detection, de-noising, and color calibration based on deep models have been explored [8].

Machine learning (ML) has been applied to image processing and classification tasks due to its excellent capability in finding hidden patterns. Well-established ML algorithms include support vector machine (SVM) which identifies an optimal hyperplane maximizing the margin, k-nearest neighbors (KNN) which finds the majority class of the K nearest neighboring data points, decision tree which constructs a tree structure representing the decisions on different conditions and the possible outcomes, and random forest which combines multiple decision trees in parallel. Deep learning (DL), a subfield of machine learning, gained significant attention for computer vision due to its feature extraction capacity. The convolutional neural network (CNN) is a feedforward neural network that consists of multiple convolution layers and pooling layers that perform feature extraction of input images and dimensionality reduction. CNN achieved high classification accuracy in various fields including medical image classification and satellite remote sensing [9,10]. Furthermore, CNN-based models have been studied for the analysis of lateral flow assay images. A simple CNN model consisting of three convolution layers was developed as a binary classifier to diagnose COVID-19 positivity or negativity [11]. Wong et al. developed a pipeline consisting of multiple CNN models to assist with auditing the lateral flow test results for SARS-CoV-2 antibody as invalid, negative, or positive [12]. Another study achieved a binary classification of HIV home test as HIV positive or negative using an SVM model and multiple CNN models including ResNet50, MobileNetV2, and MobileNetV3 [13].

Although some ML and CNN models have been developed to analyze lateral flow assay images, those models mostly focus on binary classification distinguishing between positive and negative diagnostics or use models with more complex architectures to achieve higher classification accuracy [14]. In this study, the ML and CNN models were developed to quantify the analyte load from lateral flow assay images taken in a portable box that controls the lighting condition and background. A comparative analysis was performed with a focus on light-weight models including the traditional ML and light-weight CNN for portability and affordability, which are crucial for point-of-care testing. The multi-class classification using ordinal encoding, which gives the sensitivity equivalent to the current gold standard testing, was explored to improve robustness. Furthermore, the impacts of various algorithms and the effects of the image conditions including size, colorspace, and noise level toward the quantification performance were investigated.

## 2. Materials and Methods

### 2.1. Materials

The COVID-19 Antigen Home Tests were provided by Central Infusion Alliance. Recombinant SARS-CoV-2 nucleocapsid protein (N protein) was purchased from Millipore Sigma and used in place of a specimen. Bovine Serum Albumin Standard was purchased from Thermo Fisher Scientific and used to measure the concentration of the purchased N protein. Bio-Rad Protein Assay Dye Reagent Concentrate was used for a Bradford assay, which quantifies protein concentrations, to establish the ground truth for our models.

### 2.2. Lateral Flow Assay

The concentration of SARS-CoV-2 N protein was measured using the Bradford assay by following the protocol provided by the manufacturer. The rapid test images were collected using COVID-19 Antigen Home Tests with varying loads of SARS-CoV-2 N protein. The SARS-CoV-2 N protein was added to the extraction buffer, and then the instructions included in the test kit were followed. Briefly, varying loads of SARS-CoV-2 N protein (0.074 ng, 0.18 ng, 0.37 ng, 0.74 ng, 1.8 ng, 3.7 ng, and 7.4 ng) were added to the extraction buffer, then the mixture buffer was added to the sample port and left to develop results for 15 min. Each protein load and the control (buffer without N protein) were tested in triplicate using 3 test kits, which resulted in a total of 24 test kits. Multiple photos of the test and control lines for each test kit were taken after the 15 min reaction. The control tests were conducted without the addition of SARS-CoV-2 N protein.

### 2.3. Data Collection

**Figure 1:**
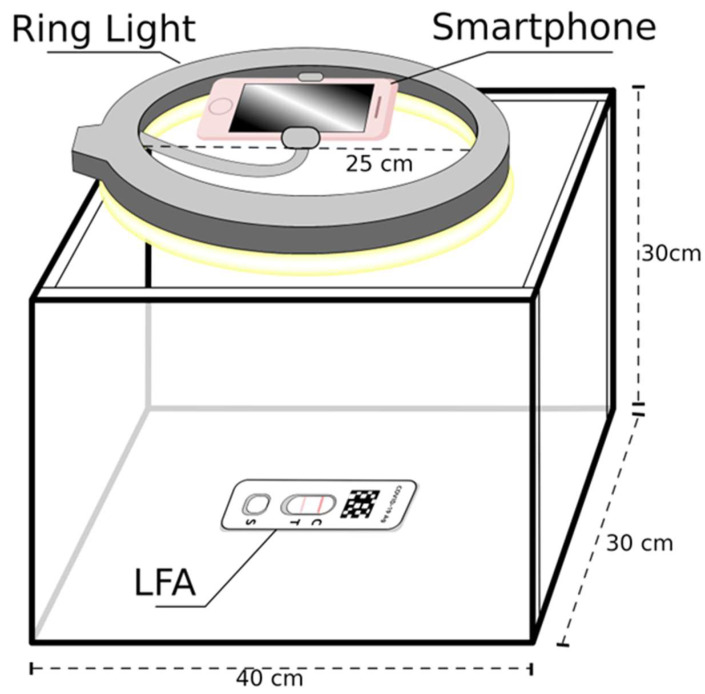
Smartphone-captured image data collection process utilized a ring light positioned overhead a white photography box containing an LFA to limit the impact of variable lighting conditions.

Using a white foam-core board free of blemishes, a 40 cm × 30 cm × 30 cm photography box [15] was constructed with an open top. A 25 cm diameter ring light positioned level with the top of the box, as seen in Figure 1, was used to provide even lighting free of shadows to control for intra- and inter-day variable lighting conditions. The rapid test kit was placed in the center of the box and photos were taken from above with an iPhone 6s Plus mobile phone camera which was attached to the center of the ring light. No flash was used. As the indicator line became less saturated, additional photos were taken. The total number of images per class after removing poor quality images unsuitable for training were 263, 302, 404, 362, 245, 362, 245, 185, 120, and 98 for control, 0.074 ng, 0.18 ng, 0.37 ng, 0.74 ng, 1.8 ng, 3.7 ng, and 7.4 ng, respectively, for a sample size of 2586. As N protein concentration reduced, class size increased to improve the models’ precision.

### 2.4. Data Preprocessing

The dataset containing the lateral flow assay images was labeled with the SARS-Cov-2 N protein loads. The images taken by smartphones will have varying magnification, angle, and lighting depending on the conditions. In order to simulate different conditions, the original images in the training dataset were augmented using ImageDataGenerator from Keras. The images were augmented by specifying the following parameters: the rotation_range at 20, zoom_range at 0.1, and brightness_range at (0.9, 1.1). The images were further normalized by setting the rescale parameter to be 1.0/255.0. The label (N protein loads) was converted to the ordinal categories using ordinal encoding. The dataset was split into 80% train data and 20% test data.

### 2.5. Machine Learning Model and Evaluation

Classification models were developed to quantify the SARS-CoV-2 N protein. Various algorithms were used to build classification models including SVM, KNN, decision tree, random forest, and CNN. The input features used for these models were the augmented images of the lateral flow assay results tested with varying loads of N protein. The known N protein loads were used as the ground truth output during training. The dataset was split into 80% training data and 20% test data. Five-fold cross-validation was applied to split the training data into 5 subsets and used 4-fold to train and 1-fold to validate the model in each iteration. The support vector classifier (SVC), KNN, decision tree, and random forest models were implemented using Scikit-Learn libraries with the default hyperparameter settings. The CNN, based on the LeNet-5 architecture [16] with slight modifications, was implemented using Keras libraries. The CNN architecture consists of 2 convolutional layers followed by an average pooling layer, 2 dense layers with ReLu activation function, and a final dense layer with softmax activation function. The CNN model was compiled using Adam optimizer with the default hyperparameters and the sparse_categorical_crossentropy as the loss function. The batch size used was 10 and the number of epochs was varied between 15 and 22. The model performance was evaluated by calculating the accuracies of all the models by comparing the predicted N protein loads and the actual N protein loads. The confusion matrix was visualized for some models.

### 2.6. Comparative Analysis

A comparative analysis including algorithm, image size, colorspace, and noise comparisons was performed to evaluate the model performances. For algorithm comparison, the SVC, KNN, decision tree, random forest, and CNN models developed using the library’s default hyperparameter were compared. Based on the performance, the random forest and CNN models were selected for further experiments. The random forest and CNN models were trained using the images resized into various sizes between 16 × 16 and 128 × 128 with the other conditions unchanged, and the model performances were compared for a size comparison study. For colorspace comparison, the images were converted into different colorspaces including RGB, HSV, YCrCb, CIE XYZ, or CIELab using openCV with the image size fixed at 128 × 128. The random forest and CNN models were trained on these converted images with the other conditions unchanged. Furthermore, the Gaussian noise with the varying standard deviation (STD) between 0 and 50 and a fixed noise mean at 0 was added to the images with the image size and colorspace fixed at 128 × 128 and RGB, respectively. The random forest and CNN models were trained on these images and the model performances were evaluated by comparing the accuracies.

Each experiment was performed ten times by varying the random state. Statistical analysis was performed using the Friedman test followed by the Nemenyi post hoc test, and the difference was considered significant at *p* < 0.05.

## 3. Results and Discussion

**Figure 2:**
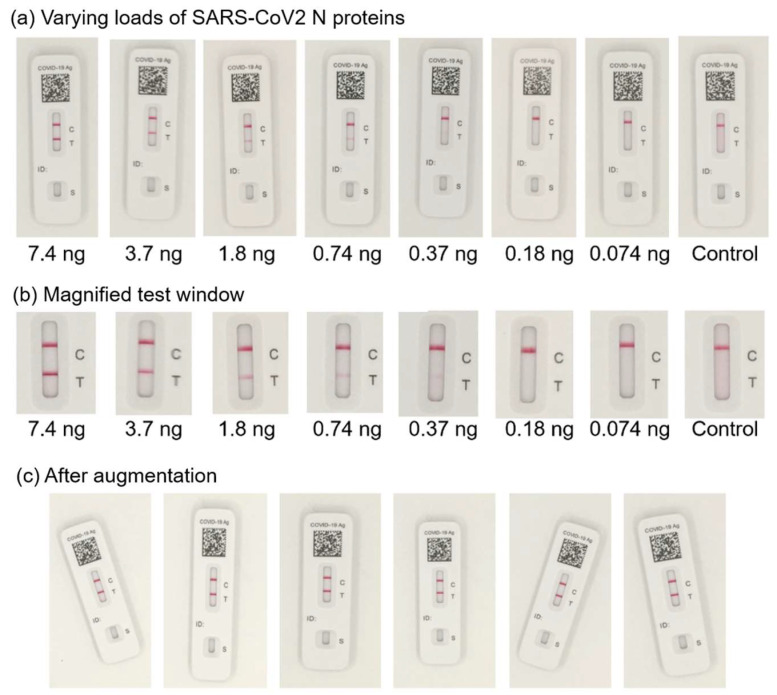
(**a**) The smartphone images of lateral flow assay test conducted with varying loads of SARS-CoV-2 N proteins. (**b**) Magnified test window. (**c**) Sample images after augmentation.

The lateral flow assay test strips have a limited detection range where the appearance of the test line correlates with the analyte. This limit of detection was established by varying the SARS-CoV-2 N protein loads. This process identified that the indicator line was not visible for two subsequent protein loads at 0.074 ng. The viral loads of infectious diseases are often expressed in log10 value. For example, the association of COVID-19 mortality and the viral load was reported based on the significant difference between the SARS-CoV-2 log10 viral load of the patients who survived and those who passed away [17]. The current gold standard in respiratory tract infection detection is also semi-quantitative [18]. Considering the sensitivity required in log10 intervals to estimate the disease severity/mortality and the detection range of the test strip, multi-class classification using ordinal encoding was employed instead of regression models, and the N-protein load range of 0.074 to 7.4 ng was used in this study. Figure 2a,b shows the lateral flow assay images tested with the N-protein load range of 0.074 to 7.4 ng. As the accuracy of the machine learning model is highly dependent on the quality of data, the smartphone images were collected in a photography box made of a white foam-core board to limit outside influences. In order to simulate the real-world images and enhance the model generalizability, multiple augmentations were applied to the images (Figure 2c).

Previous studies have shown that the variability of brightness between images collected by different models of smartphones can negatively impact the accuracy of a machine learning model [19]. To account for these varying conditions across smartphones, the dataset images were augmented with images that simulate a range of brightness, focal lengths, and signal–noise ratio.

### 3.1. Algorithm Comparison

**Figure 3:**
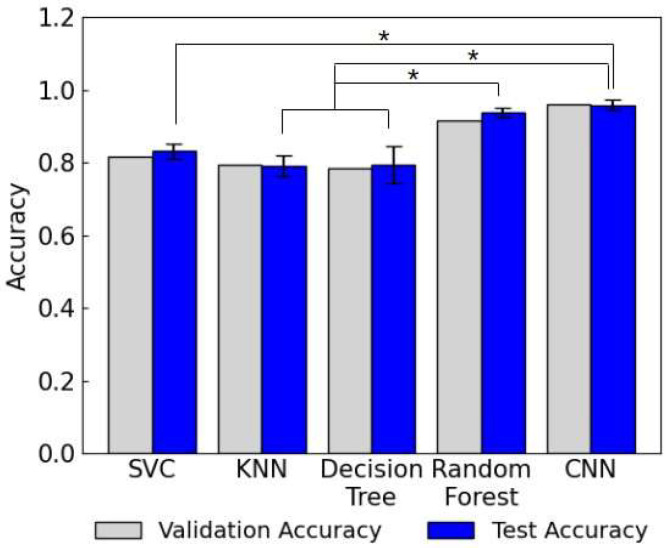
The test accuracies, which represent the rate of correct model predictions, of the SVC, KNN, decision tree, random forest, and CNN models. The asterisk indicates a statistically significant difference (*p* < 0.05).

To evaluate the classification performance of different ML and DL models, the SVM, KNN, decision tree, random forest, and CNN models were trained on the lateral flow assay images as the input and the SARS-CoV-2 N protein load as the target. The default hyperparameters from the libraries were used and the comparative analysis was performed by evaluating the performance metrics. As shown in Figure 3, the test accuracies of SVC, KNN, decision tree, random forest, and CNN models were 0.831, 0.790, 0.794, 0.937, and 0.958, respectively. All the models showed comparative accuracy during testing compared to the validation accuracy.

**Figure 4:**
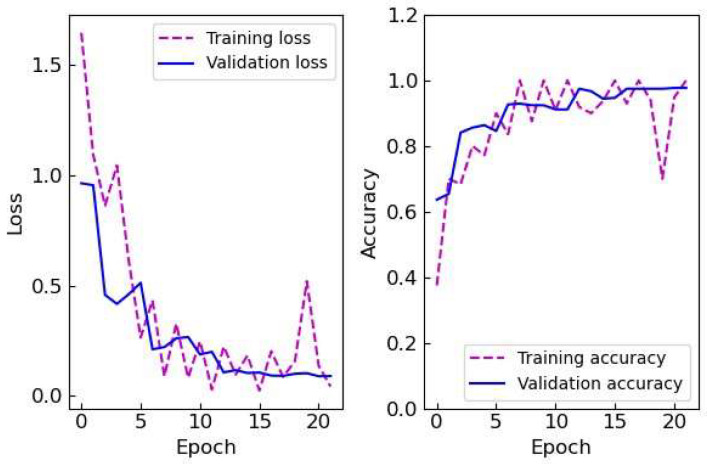
The training and validation losses and accuracies of the CNN model. The loss and accuracy represent the error of the model and the rate of correct model predictions, respectively.

The CNN model showed the highest accuracy followed by the random forest, SVC, decision tree, and KNN models with accuracies over 0.9 observed for both the random forest and CNN models. The statistical significances were observed between the SVC and CNN models, between the KNN/decision tree models and the random forest, and between the KNN/decision tree models and CNN models. There was no statistically significant difference observed between the random forest and CNN models. SVM, KNN, and decision tree are well-established ML algorithms used for various prediction and classification tasks. These traditional ML algorithms have been employed for image classification in fields such as remote sensing by improving the algorithm suitable for each task [20]. Random forest is an ensemble method that combines multiple decision trees in parallel by aggregating/voting the outcomes from each tree. It often outperforms decision tree models in prediction and classification tasks by enhancing its accuracy and preventing overfitting. The higher accuracy of the random forest model compared to the decision tree model corresponds with this trend. CNN has been widely adopted for image classification tasks due to its exceptional ability to learn from and interpret image data. Among different CNN architectures proposed for image classification tasks, LeNet-5 is a small and the most classical CNN architecture composed of two convolution layers and two average pooling layers followed by three dense layers used for the classification of handwritten images. It is capable of performing simple image classification tasks with high accuracies even with its light-weight architecture and simple implementation. The result from this comparative analysis also supports that the CNN model outperforms traditional ML models in classifying lateral flow assay images. Figure 4 shows the training and validation losses and accuracies of the CNN models with respect to the number of epochs. The losses decreased and accuracies enhanced as the number of epochs increased and plateaued after epoch 17. Therefore, the CNN model trained with epoch 17 along with the random forest model was selected for further experiments due to their higher accuracies compared to the other models.

### 3.2. Image Size Comparison

**Figure 5:**
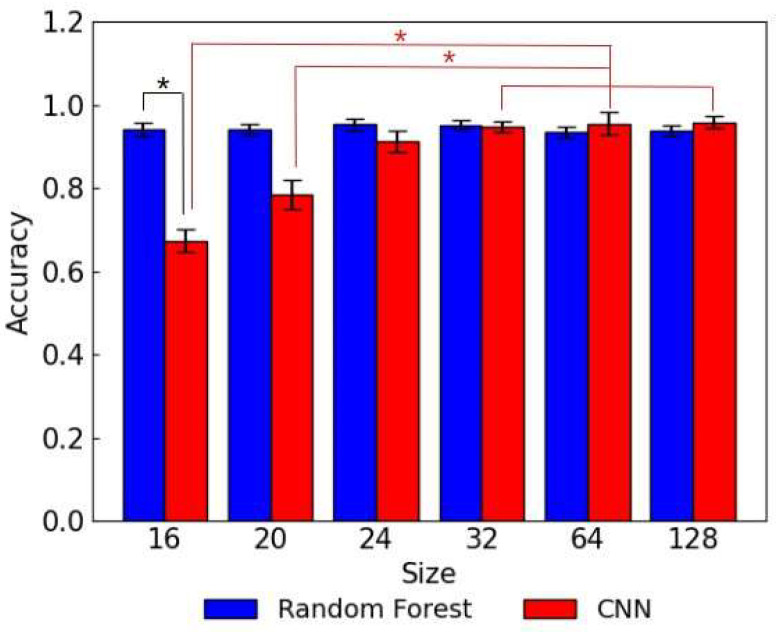
The test accuracies of the CNN models trained on the lateral flow assay images with different sizes. The asterisk indicates a statistically significant difference (*p* < 0.05).

The image size in a dataset has a significant impact on the resource requirements and processing time. Therefore, it is important to assess the size effect of lateral flow assay images on the model performance. The random forest and CNN models were developed using lateral flow assay images with varying image sizes from 16 × 16 to 128 × 128 as input. The accuracies of the random forest models trained on 16 × 16, 20 × 20, 24 × 24, 32 × 32, 64 × 64, and 128 × 128 images were 0.942, 0.940, 0.952, 0.951, 0.936, and 0.937, respectively. The accuracies of the CNN models trained on 16 × 16, 20 × 20, 24 × 24, 32 × 32, 64 × 64, and 128 × 128 images were 0.673, 0.784, 0.913, 0.948, 0.955, and 0.958, respectively (Figure 5).

**Figure 6:**
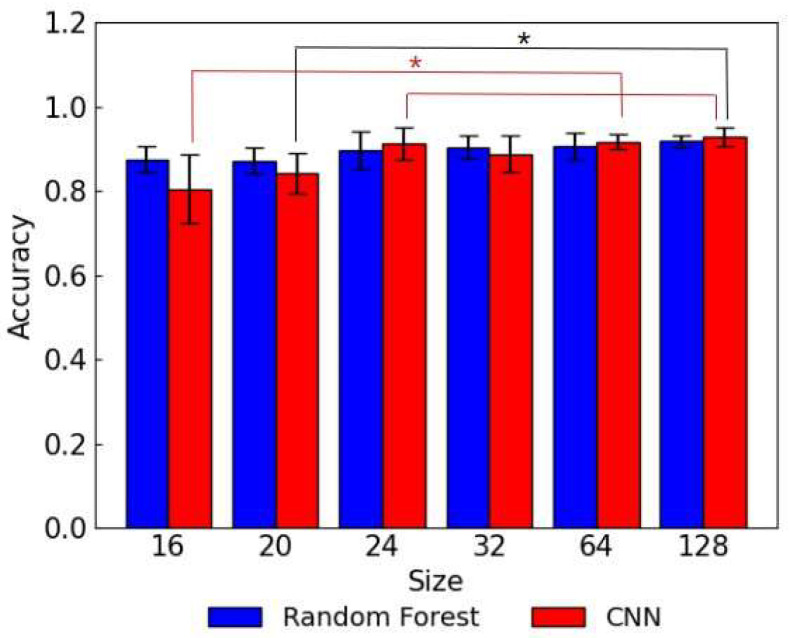
The test accuracy (breakdown by image size) of a single random forest model and CNN model trained on the lateral flow assay images with all sizes from 16 × 16 to 128 × 128. The asterisk indicates a statistically significant difference (*p* < 0.05).

The statistical significances were observed between the random forest and CNN models trained using 16 × 16 images and between the CNN models trained using 16 × 16/20 × 20 images and the CNN models trained using 32 × 32/64 × 64/128 × 128 images. As shown in Figure 5, a different trend was observed between the accuracies of the random forest models and CNN models. The random forest models exhibited high accuracies when trained on smaller images and the accuracy dropped slightly when the image sizes were increased over 32 × 32. In contrast, the accuracies of the CNN models improved significantly as the image size was increased from 16 × 16 to 24 × 24, followed by a slight increase from 24 × 24 to 64 × 64. In the study conducted by Thambawita et al., low-resolution images were reported to decrease the classification performance of CNN models significantly, and increased performance of the CNN-based model was observed when trained using higher-resolution images in endoscopy image classification [21]. The low accuracies of the CNN models trained on smaller-size images are in agreement with the previous reports. Although CNN models generally outperform traditional ML models in image-handling tasks, some ML models, especially when trained on smaller datasets, have demonstrated competitive performances with CNNs [22]. This result indicates that the random forest models are able to capture the patterns in the lower-resolution images of lateral flow assay more accurately compared to the CNN models. Furthermore, a single random forest model and a single CNN model were trained on the lateral flow assay images with all sizes from 16 × 16 to 128 × 128 combined to validate the generalizability and robustness against different image sizes. Figure 6 shows the accuracy breakdown of the random forest model and CNN model by image sizes. There was no significance observed between the accuracy breakdowns of the random forest model. In the case of the CNN model, the statistical significances were observed between the accuracy for 16 × 16 images and that for 24 × 24/64 × 64/128 × 128 images and between the accuracy for 20 × 20 and that for 128 × 128 images. By using the dataset which includes the lateral flow assay images with different sizes, the accuracy of the CNN model improved significantly for the smaller image sizes. It indicates that incorporating higher-resolution images into training data possibly enhances the accuracy of the CNN model for lower-resolution images.

### 3.3. Colorspace Comparison

The colorspace, which is a specific organization of colors representing them as numbers, is an important factor in image classification. To evaluate the impact of colorspace on the lateral flow assay image classification, the random forest and CNN models were trained using the images converted to various colorspaces including RGB, HSV, YCrCb, CIE XYZ, and CIELab. The accuracies of the models are shown in Table 1.

**Figure 7:**
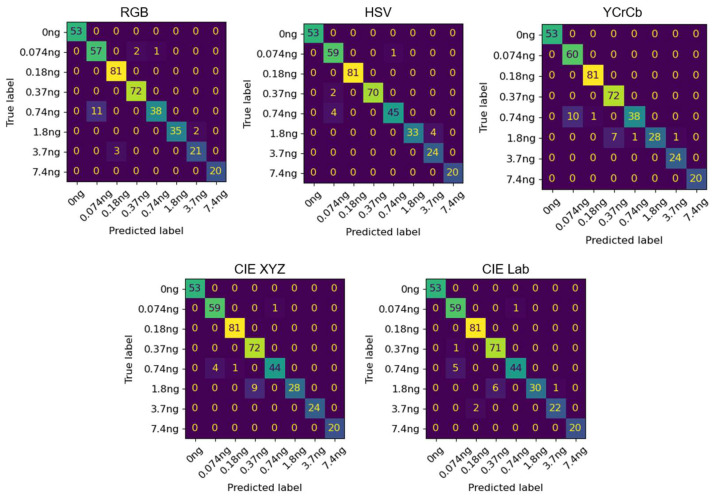
The confusion matrices of the CNN models trained on the lateral flow assay images converted into RGB, HSV, YcrCb, CIE XYZ, and CIELab colorspaces.

As shown in Table 1, all the random forest models and CNN models showed accuracies above 0.913 and 0.953, respectively. The random forest model trained on HSV-converted images exhibited the highest accuracy among the other random forest models followed by the model trained on RGB- and CIE XYZ-converted images. The CNN models trained on CIELAB-converted images as well as YCrCb- and CIE XYZ-converted images exhibited the highest accuracy among the CNN models. The HSV colorspace is commonly used to improve the outcomes of machine learning models including image segmentation and clustering [23,24], which corresponds with the results of this study. While the original RGB images provide satisfactory results at 0.937 accuracy in the random forest models, slight improvements in model outcomes can be achieved by converting training data to HSV colorspace. Similarly, slightly higher performances are expected from the CNN models by converting the images to CIELAB, YCrCb, and CIE XYZ. To further investigate the performances of the CNN models, the confusion matrices of the CNN models are shown in Figure 7. While the CNN models were able to predict all the concentrations with high precisions including 100% precision in predicting the control class, there were some misclassifications. All the models had some 0.074 ng images misclassified as the 0.74 ng class.

### 3.4. Noise Comparison

**Figure 8:**
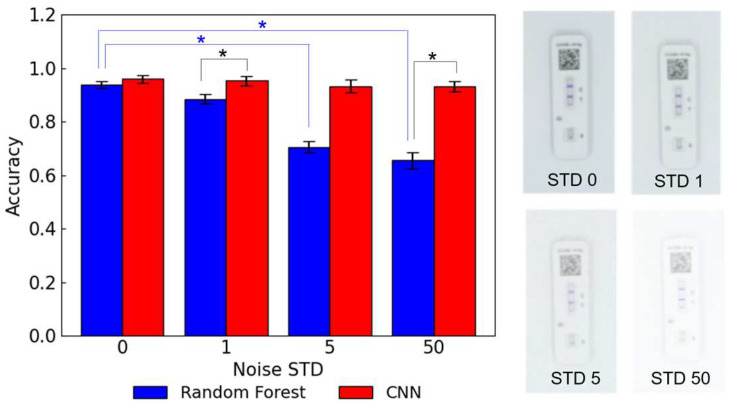
The accuracies of the random forest and CNN models trained on the images with different noise levels and the photos of the images. The asterisk indicates a statistically significant difference (*p* < 0.05).

The impact of noise levels on the model accuracy was evaluated by adding Gaussian noise with varying noise STD to the lateral flow assay images (Figure 8). The random forest and CNN models trained on the images with the noise STDs of 1, 5, and 50 were evaluated. As shown in Figure 8, the accuracies of the random forest models dropped significantly from 0.937 to 0.655 as the noise STD was increased from 0 to 50. In contrast, the accuracies of the CNN models stayed above 0.9 with a slight decrease from 0.959 to 0.931. The statistical significances were observed between the random forest models trained on images with the noise STDs of 0 and 5, between the random forest models trained with the noise STDs of 0 and 50, as well as between the random forest model and CNN model with the noise STD of 1, and between the random forest model and CNN model with the noise STD of 50. CNN often outperforms traditional ML models in image classification tasks due to its excellent capacity to identify key patterns in images. The convolution layers and pooling layers in CNN architectures perform automatic feature extraction and dimensionality reduction, which allows CNNs to learn the characteristic features of the noise while training [25]. Moreover, some CNN architectures have been developed for image denoising models [26]. The higher accuracies of the CNN models compared to the random forest models when trained on noisy images could be due to the inherent feature extraction capacity of CNNs.

## 4. Conclusions

In this study, we have successfully adapted ML and CNN methods to quantify the lateral flow assay results. ML and CNN models that classify the lateral flow assay images based on the SARS-CoV-2 load were developed to allow the quantification of lateral flow assay results using photos taken by smartphones. The comparative analysis of the models revealed that the random forest and CNN models outperformed the SVC, KNN, and decision tree models with high accuracies over 0.90. The random forest models exhibited higher accuracies compared to the CNN models when trained using small-size images. In contrast, the CNN models were more robust compared to the random forest models against the Gaussian noise on the images. Moreover, the colorspaces of the dataset images were found to influence the model performance only slightly, depending on the combination of the algorithm and colorspace. These findings demonstrate the feasibility of extracting quantitative data from a lateral flow assay using ML and CNN models. Since this is a proof-of-concept study that validates the machine learning-based quantification of the COVID-19 rapid test, further experiments should include assay results from multiple brands in the market to improve the practical significance and cross-brand generalizability.

## Figures and Tables

**Table 1:** The test accuracies and standard deviations of the random forest and CNN models trained on images converted to different colorspaces.

Colorspace	Random Forest	CNN
RGB	0.937 ± 0.014	0.958 ± 0.014
HSV	0.945 ± 0.013	0.953 ± 0.029
YCbCr	0.916 ± 0.011	0.963 ± 0.009
CIE XYZ	0.935 ± 0.013	0.961 ± 0.016
CIELAB	0.913 ± 0.010	0.969 ± 0.012

## Data Availability

The raw data utilized for this study are available on Figshare at http://doi.org/10.6084/m9.figshare.c.7581998 (accessed on 12 December 2024).
